# Supplementary material for: Predicting age of respiratory syncytial virus infection from birth timing
Source: Nat Commun. 2026 Jan 13;17:1178. doi: 10.1038/s41467-025-67947-3 (PMC12858871; doi:10.1038/s41467-025-67947-3)
Supplement: Supplementary file 2 — Reporting Summary [file 41467_2025_67947_MOESM2_ESM.pdf]

nature portfolio

Corresponding author(s): Chris G. McKennan (chm195@pitt.edu) and Tina V. Hartert (tina.hartert@vumc.org)

Last updated by author(s): Chris G. McKennan

Reporting Summary

Nature Portfolio wishes to improve the reproducibility of the work that we publish. This form provides structure for consistency and transparency in reporting. For further information on Nature Portfolio policies, see our Editorial Policies and the Editorial Policy Checklist.

Statistics

For all statistical analyses, confirm that the following items are present in the figure legend, table legend, main text, or Methods section.

n/a	Confirmed	
☐	☒	The exact sample size (*n*) for each experimental group/condition, given as a discrete number and unit of measurement
☐	☒	A statement on whether measurements were taken from distinct samples or whether the same sample was measured repeatedly
☐	☒	The statistical test(s) used AND whether they are one- or two-sided<br>*Only common tests should be described solely by name; describe more complex techniques in the Methods section.*
☐	☒	A description of all covariates tested
☐	☒	A description of any assumptions or corrections, such as tests of normality and adjustment for multiple comparisons
☐	☒	A full description of the statistical parameters including central tendency (e.g. means) or other basic estimates (e.g. regression coefficient) AND variation (e.g. standard deviation) or associated estimates of uncertainty (e.g. confidence intervals)
☐	☒	For null hypothesis testing, the test statistic (e.g. *F*, *t*, *r*) with confidence intervals, effect sizes, degrees of freedom and *P* value noted<br>*Give P values as exact values whenever suitable.*
☒	☐	For Bayesian analysis, information on the choice of priors and Markov chain Monte Carlo settings
☒	☐	For hierarchical and complex designs, identification of the appropriate level for tests and full reporting of outcomes
☐	☒	Estimates of effect sizes (e.g. Cohen's *d*, Pearson's *r*), indicating how they were calculated

Our web collection on statistics for biologists contains articles on many of the points above.

Software and code

Policy information about availability of computer code

Data collection	No computer code was used to collect the data used in this study.
Data analysis	All code, as well as instructions on how to run it, has been uploaded to https://github.com/chrismckennan/RSV-Infection-Age. Since we cannot share birth dates, we have provided tools to simulate birth dates and RSV infection ages, which can then be used to estimate model parameters.

For manuscripts utilizing custom algorithms or software that are central to the research but not yet described in published literature, software must be made available to editors and reviewers. We strongly encourage code deposition in a community repository (e.g. GitHub). See the Nature Portfolio guidelines for submitting code & software for further information.

Data

Policy information about availability of data

All manuscripts must include a data availability statement. This statement should provide the following information, where applicable:

- Accession codes, unique identifiers, or web links for publicly available datasets
- A description of any restrictions on data availability
- For clinical datasets or third party data, please ensure that the statement adheres to our policy

RSV circulation data used in this study were obtained from the National Respiratory and Enteric Virus Surveillance System (NREVSS). The data can be requested from NREVSS at nrevss@cdc.gov. The raw data from the INSPIRE cohort used to estimated model parameters, as well as the raw data from the COAST and URECA

cohorts used to test the estimated model, contain identifiable information such as birth dates, and therefore cannot be made publicly available. Investigators may submit an asthma- or allergy-related application to use these data (https://cadre.med.wisc.edu). The process generally takes 1-2 months. Data from the PRIMA cohort cannot be shared as per our contract and data use agreement with TennCare.

Research involving human participants, their data, or biological material

Policy information about studies with human participants or human data. See also policy information about sex, gender (identity/presentation), and sexual orientation and race, ethnicity and racism.

Reporting on sex and gender	Sex (male/female) was self-reported. All analyses were done using data from both sexes. Sex was included as a covariate when analyzing RSV surveillance data from the INSPIRE, URECA, and COAST cohorts.
Reporting on race, ethnicity, or other socially relevant groupings	Race (White or Non-white) was used as a covariate when analyzing RSV surveillance data from the INSPIRE, URECA, and COAST cohorts because it explained variation in and was useful in predicting RSV infection time.
Population characteristics	All population characteristics can be found in Table 1 of the manuscript.
Recruitment	The recruitment of study participants has been previously reported (see references 8-11 in our manuscript). We reference these citations in the first paragraph of Results. We further note that INSPIRE is a population-based cohort whose participants had birth dates between June and December, as these are the children most at risk of infection during the first year. We discuss this in the Discussion section of our manuscript.
Ethics oversight	Studies in INSPIRE, URECA, and COAST were approved by the Institutional Review Board of Vanderbilt University Medical Center, the University of Wisconsin and Western Institutional Review Board, and the Human Subjects Committee at the University of Wisconsin-Madison, respectively. Studies in PRIMA were approved by the Kaiser Permanente Northern California and Vanderbilt University Institutional Review Boards.

Note that full information on the approval of the study protocol must also be provided in the manuscript.

Field-specific reporting

Please select the one below that is the best fit for your research. If you are not sure, read the appropriate sections before making your selection.

☒ Life sciences ☐ Behavioural & social sciences ☐ Ecological, evolutionary & environmental sciences

For a reference copy of the document with all sections, see nature.com/documents/nr-reporting-summary-flat.pdf

Life sciences study design

All studies must disclose on these points even when the disclosure is negative.

Sample size	Samples sizes in the INSPIRE, URECA, and COAST cohorts were determined according to their cohort designs (see references 8-11 in the main text). We used all samples with a known RSV infection status (infected or not) by age one year. The non-birthdate covariates race (White or Non-White), daycare attendance in the first year of life (yes or no), older siblings (yes or no), and sex (male or female) were included in our model to help predict age of first RSV infection. Please see "Incorporating additional covariates into the model" in Methods for further details.
Data exclusions	Subjects were only excluded in the INSPIRE, URECA, and COAST cohorts if they did not have a known RSV infection status (infected or not) by age one year.
Replication	Our model was estimated in the INSPIRE cohort. We used URECA and COAST as replication by predicting RSV infection in these cohorts. Replication was successful.
Randomization	No randomization was done. Infection was natural, not assigned.
Blinding	Researchers were blinded to infection status during sample collection, as it was unknown prior to collection. Analysis were not blinded, as infection status was the outcome in statistical models.

Reporting for specific materials, systems and methods

We require information from authors about some types of materials, experimental systems and methods used in many studies. Here, indicate whether each material, system or method listed is relevant to your study. If you are not sure if a list item applies to your research, read the appropriate section before selecting a response.

Materials & experimental systems

n/a	Involved in the study
☒	☐ Antibodies
☒	☐ Eukaryotic cell lines
☒	☐ Palaeontology and archaeology
☒	☐ Animals and other organisms
☒	☐ Clinical data
☒	☐ Dual use research of concern
☒	☐ Plants

Methods

n/a	Involved in the study
☒	☐ ChIP-seq
☒	☐ Flow cytometry
☒	☐ MRI-based neuroimaging

Plants

Seed stocks	N/A
Novel plant genotypes	N/A
Authentication	N/A
